# Supplementary material for: The Efficacy of S-1 as Adjuvant Chemotherapy for Resected Biliary Tract Carcinoma: A Propensity Score-Matching Analysis
Source: J Clin Med. 2021 Mar 1;10(5):925. doi: 10.3390/jcm10050925 (PMC7957643; doi:10.3390/jcm10050925)
Supplement: Supplementary file 1 [file jcm-10-00925-s001.zip › jcm-1090350_supplementary 2_revised_20210303_Final.docx]

**Table** **A1.** Profiles and tumour characteristics of the patients with perineural invasion in each group of the matched cohort.

|  |  | S-1 group |  | Observation group |  | *p* value |
| --- | --- | --- | --- | --- | --- | --- |
|  |  | *n* = 29 |  | *n* = 27 |  |  |
| Age [y] | | 71 (52-82) |  | 74 (59-83) |  | 0.096 |
| Gender, male | | 21 (72) |  | 17 (63) |  | 0.449 |
| Diagnosis | |  |  |  |  |  |
|  | Hilar cholangiocarcinoma | 10 (34) |  | 10 (37) |  | 0.811 |
|  | Distal cholangiocarcinoma | 17 (59) |  | 14 (52) |  |  |
|  | Gallbladder carcinoma | 2 (7) |  | 3 (11) |  |  |
| Serum CA19-9 [U/ml] | | 71 (1-1807) |  | 102 (21-2524) |  | 0.125 |
| Hepatectomy | | 10 (34) |  | 11 (41) |  | 0.629 |
| Clavien-Dindo classification, III-V | | 14 (48) |  | 12 (44) |  | 0.774 |
| Pathological findings | |  |  |  |  |  |
|  | Tumor differentiation, well | 10 (34) |  | 9 (33) |  | 0.928 |
|  | Lymphatic invasion | 21 (72) |  | 20 (74) |  | 0.889 |
|  | Venous invasion | 21 (72) |  | 25 (93) |  | 0.049* |
|  | T status, T3 and T4 | 18 (62) |  | 19 (70) |  | 0.512 |
|  | N status, N1 | 13 (45) |  | 13 (48) |  | 0.803 |
|  | R status, R0 | 22 (76) |  | 18 (67) |  | 0.447 |

* Statistical significance (*P* < 0.050).

Values in parentheses are the percentages for categorical data or range for continuous data.

CA19-9, carbohydrate antigen 19-9.
