# Supplementary material for: The Efficacy of S-1 as Adjuvant Chemotherapy for Resected Biliary Tract Carcinoma: A Propensity Score-Matching Analysis
Source: J Clin Med. 2021 Mar 1;10(5):925. doi: 10.3390/jcm10050925 (PMC7957643; doi:10.3390/jcm10050925)

**Figure S1.** Kaplan–Meier curves of recurrence-free survival (a) and overall survival (b) in the entire cohort. There was not significantly different between the S-1 and observation groups in either recurrence-free survival (26.5 vs. 56.2 months, *p* = 0.395) or overall survival (not reached vs. 86.7 months, *p* = 0.643).


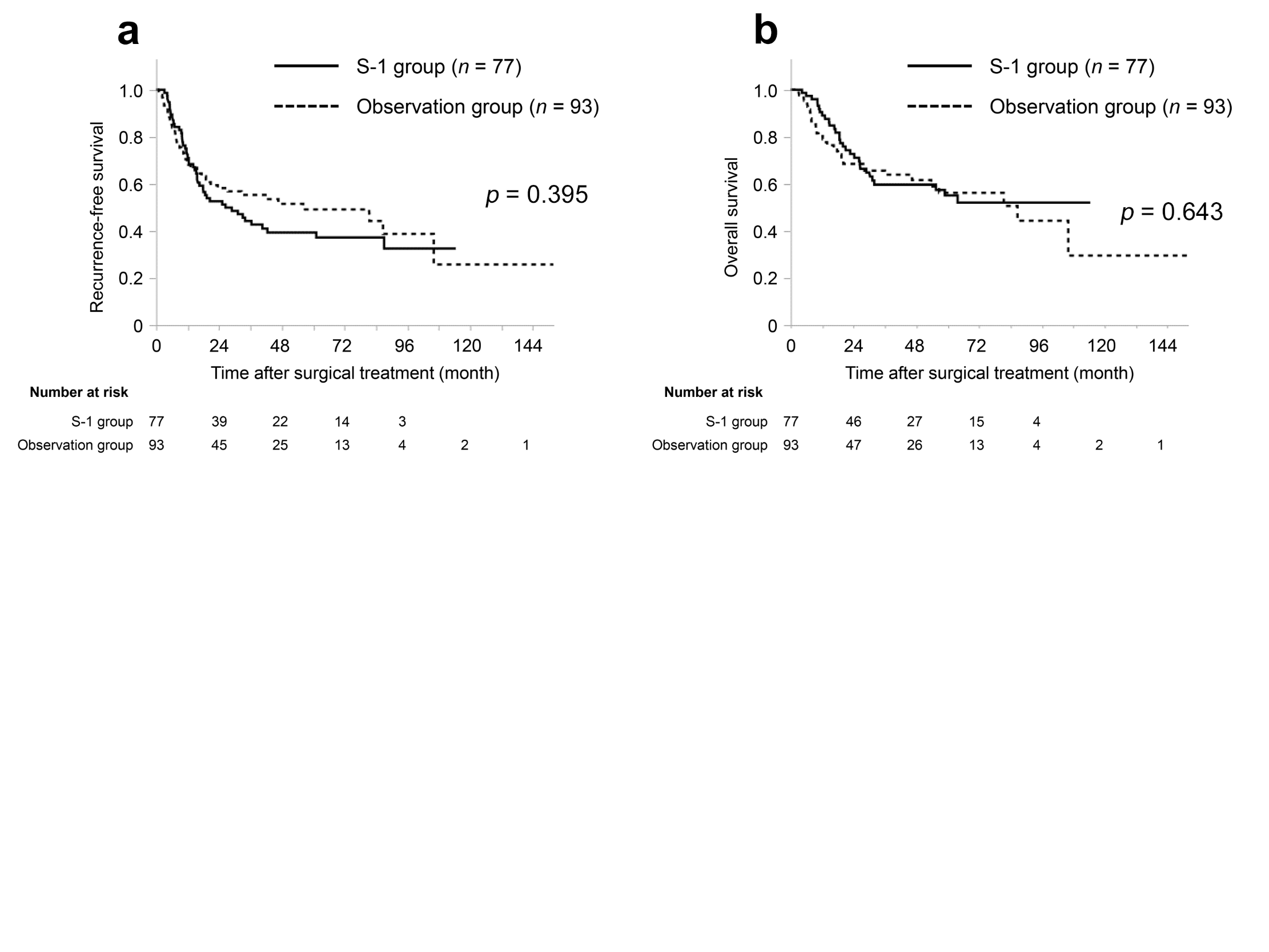


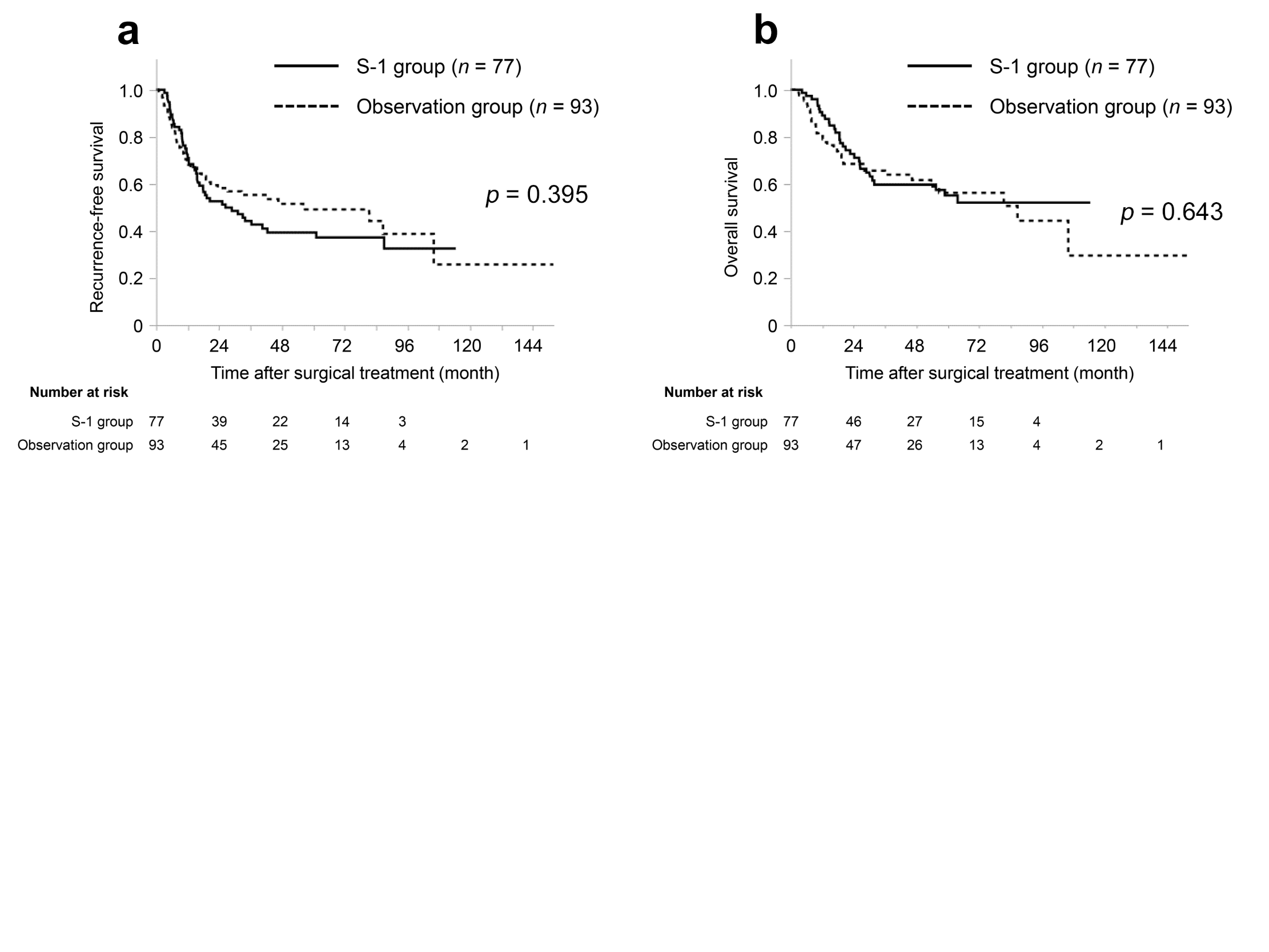

Supplement: Supplementary file 1 [file jcm-10-00925-s001.zip › jcm-1090350_supplementary 1_revised_20210303_Final.docx]
